# Molecular Pharmacology in Diabetes

**DOI:** 10.3390/ijms25053051

**Published:** 2024-03-06

**Authors:** Flávio Reis, Rosa Fernandes

**Affiliations:** 1 Coimbra Institute for Clinical and Biomedical Research (iCBR), Faculty of Medicine, University of Coimbra, 3000-548 Coimbra, Portugal; 2 Institute of Pharmacology and Experimental Therapeutics, Faculty of Medicine, University of Coimbra, 3000-548 Coimbra, Portugal; 3 Center for Innovative Biomedicine and Biotechnology (CIBB), University of Coimbra, 3004-531 Coimbra, Portugal; 4 Clinical Academic Center of Coimbra (CACC), 3004-561 Coimbra, Portugal; 5 Association for Innovation and Biomedical Research on Light and Image (AIBILI), 3000-548 Coimbra, Portugal

This Special Issue highlights the key molecules and molecular signaling pathways associated with diabetes and its multifaceted complications. Diabetes stands as a paramount global public health challenge. Inadequately managed diabetes can exert profound and far-reaching impacts on various organs and tissues within the body, including the kidneys, ocular surface, retina, and heart. Although progress has been made in therapeutic approaches to reducing glucose levels, certain complications of diabetes remain irreversible and resistant to prevention. For a comprehensive understanding of the disease, it is imperative to deepen the knowledge of the molecular signaling pathways underlying the development of diabetes and its associated complications. 

Dopamine, beyond its traditional role as a neurotransmitter, plays a crucial role in fine-tuning the control of insulin secretion, adding a layer of complexity to our understanding of the molecular mechanisms governing glucose homeostasis. While D-glucose, along with specific amino acids and fatty acids, serves as the primary physiological stimulus for insulin secretion, numerous other molecules, including gut-derived peptide hormones and classical neurotransmitters, contribute to the nuanced control of net insulin production. Among these neurotransmitters, dopamine emerges as a significant factor, acting directly on β-cells and influencing other target tissues such as the liver and skeletal muscle. The intricate interplay of these molecules serves as amplifying agents that enhance signals generated by the β-cell glucose sensing apparatus. Chaudry et al. have suggested an intriguing concept: dopamine potentially serves as an anti-incretin in pancreatic β-cells by activating the dopamine type 2 receptor (D2R). According to their proposal, this activation leads to the inhibition of insulin secretion stimulated by glucose and glucagon-like peptide 1 (GLP-1) [1]. One of the studies in this Special Issue investigates the regulatory role of dopamine in insulin signaling, glucose uptake, and catabolic activity in the liver and white adipose tissue (WAT). This research focuses on the impact of nutritional cues on dopamine secretion, particularly following a mixed meal and glucose intake in Wistar rats. In a preclinical model of type 2 diabetes, the high-fat-diet-fed diabetic Goto-Kakizaki rats, sleeve gastrectomy is shown to upregulate dopaminergic machinery, emphasizing the influence of the gut on dopamine signaling in WAT. Treatment with the dopamine agonist bromocriptine increases the GLP-1 receptor (GLP-1R) in WAT, revealing dopamine’s role in regulating GLP-1R. Interestingly, the GLP-1 receptor agonist Liraglutide has no impact on dopamine receptors. Crosstalk between GLP-1 and dopamine is demonstrated in rat WAT explants, where dopamine enhances GLP-1-induced AMPK activity in mesenteric WAT. Human WAT analysis reveals a correlation between dopamine receptor 1 (D1DR) and GLP-1R expression. The findings suggest a link between dietary and gut regulation of plasma dopamine, influencing WAT to modulate GLP-1 action. These insights into dopamine’s dual role in the pancreas and WAT may open new therapeutic avenues for enhancing metabolic control in metabolic disorders. 

Insights into the intricate molecular and cellular dysregulation of innate immunity in diabetes are given in one of the studies in this Special Issue, focusing specifically on the ocular surface and retina, and the potential interconnectedness of these mechanisms is elucidated. The innate immune system, a paramount defender against pathogens, assumes a pivotal role in the pathophysiology of diabetes. In individuals with diabetes, chronic low-grade inflammation disrupts immune homeostasis, influencing the function of immune-competent cells and the release of inflammatory mediators. While discussions on the ocular complications of diabetes often spotlight diabetic retinopathy, this manuscript underscores the often-neglected impact on the ocular surface, where inflammatory changes may occur, affecting the delicate balance of the eye’s immune privilege. Diabetic retinopathy, a well-recognized complication of diabetes, extends beyond its vascular manifestations. Inflammatory and neurovascular factors play a key role in the progression of diabetic retinopathy. The intricate network of innate immune cells in the retina faces challenges in maintaining immune homeostasis under the inflammatory milieu associated with diabetes. While substantial progress in understanding the mechanisms underlying the ocular complications of diabetes has been made through preclinical models, it is crucial to acknowledge the notable differences between rodents and human physiology that can impact the localization and regulation of the immune response in the eye. An awareness of these distinctions is imperative for advancing our comprehension of how diabetes affects the ocular immune system and tailoring potential interventions for human applications.

As therapeutic strategies for diabetes management progress, there is a growing recognition of the need for a more comprehensive and holistic approach to tackle complications associated with diabetes. The other contributions in this Special Issue explore or offer insight into promising therapeutic interventions, including novel pharmaceuticals and bioactive compounds, aiming to alleviate the impact of diabetes-related complications.

Inonotus obliquus, commonly known as Chaga mushroom, is widely used as medicinal and health food in various cultures, including China, Russia, Korea, and several Western countries. Among its extracts, Inonotus obliquus contains several compounds, including polysaccharides, triterpenes, and polyphenols, with interesting properties, such as antioxidant, anti-inflammatory, anti-glycemic, and anti-hyperlipidemic activities [2]. As diabetes nephropathy (DN) is a prevalent cause of end-stage renal disease globally, and therapeutic options for chronic renal disease (CKD) are limited, after water–ethyl acetate separation from Inonotus obliquus ethanol crude extract (EtCE-EA) from Chaga mushrooms, the potential renoprotective effects of the ethyl acetate layer in mice with DN induced by a combination of streptozotocin and unilateral nephrectomy were explored. The EtCE-EA treatment effectively regulates the blood glucose levels, albumin–creatinine ratio, and the serum creatinine and blood urea nitrogen (BUN) levels, demonstrating an improvement in renal damage. A reduction in the expression of TGF-β and α-SMA with increasing concentrations of EtCE-EA suggests a potential slowdown in the degree of kidney damage. The findings propose that EtCE-EA may offer renoprotection in diabetic nephropathy by mitigating the expression of transforming growth factor-β1 and α-smooth muscle actin.

Scientific literature supports curcumin’s anti-diabetic effects and its ability to alleviate complications associated with diabetes through diverse molecular mechanisms of action [3]. Curcumin, the active compound in *Curcuma longa* L., exhibits several physiological and pharmacological benefits, including antioxidant, anti-inflammatory, and antidiabetic properties. The former activity is related to its potent abilities to suppress oxidative stress and inflammation, protecting against diabetes-induced endothelial dysfunction and down-regulation of nuclear factor-kappa B. The compound regulates blood glucose and glycosylated hemoglobin levels in diabetic rats by modulation of the polyol pathway [4]. A novel curcuminoid was synthesized with the aim of enhancing the anti-inflammatory and antioxidant potential of curcumin while reducing the required therapeutic dose. In vitro studies show that curcumin and curcuminoid have significant anti-inflammatory effects. In vivo experiments on Goto-Kakizaki rats reveal that the curcuminoid, administered at a lower equimolar dose than curcumin, exhibits a marked hypoglycemic effect, accompanied by increased levels of glucose transporter type 4 (GLUT4) in adipose tissue. Furthermore, both compounds improved nitric oxide (NO)-dependent vasorelaxation. However, only the curcuminoid showed an enhanced response to ascorbic acid, indicating a more significant reduction in vascular oxidative and nitrosative stress. Notably, the curcuminoid enhances the antioxidant defenses in epididymal adipose tissue (EAT) and the heart. The curcuminoid demonstrates superior efficacy at a lower dose compared to curcumin, mitigating the limited therapeutic potential of curcumin and circumventing the adverse effects associated with high dosages.

One of the papers in this Special Issue reviews the evidence related to the potential of glucocorticoid (GC) metabolism as a therapeutic target for metabolic diseases. These steroid hormones secreted by the adrenal cortex are chief regulators of carbohydrate, lipid, and protein metabolism and might be involved in the development of cardiometabolic diseases (e.g., obesity, metabolic syndrome, type 2 diabetes mellitus, among others) due to the impaired regulation of their levels. The local availability of cortisol and corticosterone to activate the glucocorticoid receptor is controlled by 11β-hydroxysteroid dehydrogenase type I (11β-HSD1), which makes this enzyme and its putative inhibitors appealing targets for novel pharmacotherapy directed toward these disorders. Before major progress on this topic can be achieved, it will be important to clarify whether the reduction in the increased cortisol levels found in several cardiovascular and metabolic diseases affect the course of these disorders. Knockout mice lacking the 11β-HSD1 gene presented with a lower triglyceride concentration and increased HDL cholesterol levels, and were resistant to hyperglycemia and visceral obesity induced by a high-fat diet and stress conditions [5,6]. The use of 11β-HSD1 inhibitors can cause a reduction in the GC content independent of the hypothalamic–pituitary–adrenal axis (which regulates the circulating GC levels) due to the control of cell and tissue processes. These promising preclinical results, namely in animal models, as well as in some placebo-controlled, randomized trials, deserve validation in large clinical trials, in order to confirm whether this enzyme could be a good novel therapeutic target for diabetes and other cardiometabolic diseases.

Another potential therapeutic target for diabetes is discussed in a paper included in this Special Issue. Glycosphingolipids (GSLs), a major group of lipids containing a backbone of sphingoid bases and a polar sphingosine head, have been associated with the development of diabetes due to their participation in several metabolic processes, including lipid metabolism, insulin sensitization, oxidative stress, and inflammation, among others. This paper reviewed preclinical evidence of the importance of these mechanisms in several animal models of diabetes, focusing on three main topics: the participation of a monosialodihexosylceramide (GM3) in the interaction between insulin and its receptor; the putative role played by ceramide (Cer) and lactosylceramide (LacCer) in mitochondrial dysfunction and apoptosis; and the involvement of LacCer in mechanisms of oxidative stress and inflammation. Preclinical data suggesting a pro-diabetic competitive role of GM3 with the insulin–insulin receptor interaction, obtained both from in vitro and from mouse models of diabetes, need to be confirmed using GM3 synthase-specific inhibitors. In addition, under conditions of hyperglycemia, increased levels of LacCer can contribute to oxidative stress, a major driver for diabetes and its cardiovascular complications. Using experimental animal models of diabetes (namely the db/db mice), inhibitors of glycosphingolipid synthesis (such as the biopolymer-encapsulated D-Threo-1-phenyl-2-decanoylamino-3-morpholino-1-propanol: D-PDMP) showed the ability to reduce the LacCer content, the blood level of bulk lipids and lipoproteins, as well as the blood glucose levels, body weight, and biomarkers of oxidative stress and inflammation [7]. Further studies with advanced omics technologies (e.g., lipidomics and genomics, among others) are still needed to understand the possibility of using sphingolipid signaling networks as a therapeutic target for diabetes.

Concerning novel targets for the prevention of the progression of diabetic complications, namely diabetic foot ulcers (DFUs), a paper in this Special Issue reviews the evidence regarding heme oxygenase-1 (HO-1), which is a rate-limiting enzyme in heme degradation, generating carbon monoxide (CO), biliverdin (BV), which is converted into bilirubin (BR), and iron. This enzyme is not only a powerful antioxidant but also exerts anti-inflammatory, proliferative, angiogenic, and cytoprotective properties, which collectively could be pivotal in preventing diabetic wound healing, a major complication of diabetes and whose complex pathophysiology involves oxidative stress, inflammation, immune cell impairment, together with reduced/delayed re-epithelialization and angiogenesis. Modulators of HO-1 biosynthesis and inhibitors of HO-1′ enzymatic activity have been assessed as a potential pharmacotherapy to ameliorate diabetic wound healing. In preclinical studies, using in vitro systems and animal models, increased HO-1 expression via the activation of transcription factors or gene therapy, as well as the use of natural or pharmacological inducers to increase HO-1 enzymatic activity, showed antioxidant and inflammatory properties. Hemin is an inducer of HO-1 that has been approved for acute intermittent porphyria treatment but that has also exhibited beneficial effects on diabetic wound healing in animal models [8], being a potential drug candidate for DFU treatment. Another promising option as a repurposing therapy is dimethyl fumarate (DMF), a drug used for multiple sclerosis treatment that seems to be able to ameliorate diabetic wound healing [9]. DMF activates Nrf2, which regulates multiple effector enzymes, including HO-1. Safe and effective Nrf2 activator compounds should be developed for potential clinical use in diabetic patients with a high risk of developing DFUs. Other drug candidates for diabetic wound healing treatment, with proven beneficial effects in animal models, include BV/BR-based therapies and the direct administration of CO via inhalation or CO-releasing molecules [10,11]. However, further research is still needed to evaluate their efficacy and safety when exogenously used as a pharmacotherapy for DFUs.
